# What’s in a number: Falling on the sword of cut-off points and reference limits?

**DOI:** 10.4102/jcmsa.v3i1.148

**Published:** 2025-06-30

**Authors:** Verena Gounden, Nareshni Moodley

**Affiliations:** 1 Department of Clinical Biochemistry, Galway University Hospital, Newcastle Road, Galway, Ireland; 2 Department of Chemical Pathology, National Health Laboratory Service, Inkosi Albert Luthuli Central Hospital, Durban, South Africa; 3 Discipline of Chemical Pathology, School of Laboratory Medicine and Medical Sciences, Faculty of Laboratory Medicine, University of KwaZulu-Natal, Durban, South Africa

**Keywords:** reference intervals, clinical decision limits, interpretation, laboratory, biological variation

## Abstract

The marked increase in laboratory test volumes and costs internationally emphasises the need for demand management. One way that this can be implemented is by reducing unnecessary repeat testing with the provision of appropriate decision cut-off points (clinical decision limits [CDLs]) or reference intervals (RIs) with subsequent correct interpretation of laboratory results. The derivation of RIs and CDLs are fraught with technical and biological challenges. There is difficulty in conducting labour-intensive, costly, long, and complex studies, which require healthy volunteers that represent things such as different age groups, genders, races, and alternate states of health (e.g. pregnancy) within the population. It is also inappropriate to apply RIs or cut-off points from other populations, which is often what occurs when manufacturer-expected values are used. Lack of standardisation of international guidelines for CDLs and analytical methods poses a further problem. The effect of analytical and biological variation on results is also essential to consider when interpreting results. These make ideal RIs and CDLs difficult to attain and implement despite their critical need.

## Introduction

The central basis of interpretation of quantitative numerical laboratory testing, particularly in biochemistry, which contributes to the majority of testing requests, is the comparison of the patients’ results to a clinical decision limit (CDL) or reference interval (RI). All laboratory users need to have context as to how these intervals and decision limits are derived and the uncertainty around the number generated by a laboratory test.

## Reference intervals and clinical decision limits

In order for the clinician to adequately interpret a patient’s result, it is essential for the laboratory to provide a defined range of values – the RI for comparison or a cut-off point value (CDL) associated with disease or the pathological state. Appropriate interpretation of the laboratory result would facilitate the safe and effective management of the patient or individual.

The RI usually comprises 95% of the test results obtained from a presumed healthy population. This implies that 2.5% of healthy people (1 in 40) will have test results outside the RI. There is significant complexity in the process of deriving an RI. The difficult and expensive process of determining adequate RIs means that most individual laboratories are unable to derive their own RIs. RIs should be created by utilising a statistically sufficient group (a minimum of 120) of healthy reference subjects. The Clinical and Laboratory Standards Institute (CLSI) guideline does acknowledge that ‘Health is a relative condition lacking a universal definition. Defining what is considered healthy becomes the initial problem in any study …’. In addition, some of the selected participants might have subclinical disease. Furthermore, participant recruitment and getting informed consent maybe costly, time-consuming, and challenging. Participants need to cover different age groups (e.g., paediatric and geriatric patients), rare samples (e.g., cerebrospinal fluid), timed collections, dynamic function testing and serial analysis. Once selection and obtaining participants has been finalised, selection of the appropriate statistical technique needs to be considered. Choice of different techniques, for example parametric, transformed parametric, nonparametric, can affect the RIs generated. Data are often multimodal or asymmetrically distributed, with required partitioning of test subjects by sex, age, race, and other factors, which add to the complexity of the process.^1^

Many medical laboratories utilise RIs largely derived from American and European populations that may not be appropriate for the local population served by the lab.^2^ This may be acceptable for some tests but not for others. For example, Vitamin D serum levels vary in individuals, where factors such as geography and race can play a more significant role.^3^ In these instances decision limits based on clinical outcomes would be useful.

Advances in computing technology have allowed for the derivation of RIs using indirect methods via databases of laboratory results. However, this is still a complex procedure requiring adequate data mining and statistical knowledge. An advantage is that the data are readily available with resultant time and cost savings. Studies utilising this approach were able to report clinically relevant and useful RIs. These used various complex filters to eliminate results from ‘unhealthy’ subjects. Data for the derivation of the RI would include results from both hospitalised patients, patients from outpatient departments and from the general population attending their primary care provider. The majority of these studies used complex statistical algorithms for derivation of the final RIs. Current guidelines do not recommend these methods as an initial approach for establishing RIs as there is a possibility that a significant proportion of the data might not actually originate from true healthy individuals.^1^

Reference interval data for regions in Africa were analysed in a systematic review by Price et al.^4^ in 2022, where they searched PubMed for RIs from Africa published since 2010, focussing on clinical analytic chemistry, haematology and immunological parameters. Data from adults, adolescents, children, pregnant women, and the elderly were included, with exclusion of manuscripts reporting data from persons with conditions that may not classify them as healthy. Of the 179 identified manuscripts, 80 were included in this review, covering 20 countries with the largest number of studies in Ethiopia (*n* = 23, 29%). Most studies considered healthy, non-pregnant adults (*n* = 55, 69%). Nine (11%) studies included pregnant women, 13 (16%) included adolescents and 22 (28%) included children. There is a scarcity of published, thoroughly conducted RI data available from sub-Saharan Africa with insufficient regional heterogeneity (almost one third of studies were from Ethiopia). Many studies had limited the appropriateness of use of their derived RIs because of the presence of bias. Price et al. highlighted issues identified with the studies reviewed. This included the majority of studies being under-representative of groups in the local population (e.g., the elderly) or had inconsistent definitions of health. Moreover, almost half of the studies did not cite the CLSI guidelines as their reference point for the method of RI derivation. The parameters measured were varied, with different analytical platforms utilised. Almost all studies agreed that regionally appropriate RIs are important, but many did not contain strong, reliable evidence for this.^4^

Unlike RIs, cut-off points or CDLs are associated with a risk of specific adverse outcomes. Commonly used examples of CDLs include lipid parameters, glucose, haemoglobin A1c (HbA1c), and cardiac markers such as Troponin, to determine the risk of disease, to diagnose or to treat. These decision limits may be based on findings of clinical studies or expert consensus.^5^ The seemingly simple plasma glucose CDLs for the diagnosis of diabetes mellitus (DM) is perhaps a textbook example of the complexities and caveats around deriving CDLs. In 1979, the American National Diabetes Data Group recommended one set of criteria for the diagnosis of DM. These were based on the results of three relatively small studies in which cohorts of individuals without diabetic retinopathy were subjected to oral glucose tolerance tests and then followed for a period of 3–8 years to determine which patients developed retinopathy. The World Health Organization (WHO) adopted some slightly modified criteria in 1980. In 1997, the American Diabetes Association (ADA), via an expert panel, revisited these criteria and decided to lower fasting plasma glucose (FPG) from ≥ 7.8 mmol/L to ≥ 7.0 mmol/L. At the time the decision was made to retain ≥ 11.1 mmol/L as the 2-h oral glucose tolerance test (OGTT) value to prevent disruption, as many large epidemiological studies had used this value to define diabetes. Several studies have also demonstrated that these existing cut-offs for FPG and 2-h OGTT glucose (OGTTG) are not equivalent in the diagnosis of diabetes. The same patient may at the same time have an elevated 2-h OGTTG while having ‘normal’ FPG. Furthermore, both the ADA (< 5.6 mmol/L) and WHO (< 6.1 mmol/L) have different decision limits for what is considered a normal fasting glucose.^6^ Throw into the mix, HbA1c as a diagnostic test for DM, with its method-dependent issues with haemoglobin variants, affectation by common disorders such as iron deficiency, etc., and the waters can seem even muddier. There is no safe level of euglycaemia that is biologically viable at which microvascular changes will never occur.^7^

The 99th percentile upper reference limit of cardiac troponin has been utilised for over 20 years as an assay-specific threshold for the diagnosis of myocardial infarction however there is no standardisation in the calculation and reporting of the 99th percentile. Assay imprecision can affect thresholds defined and more significantly the ‘healthy’ population used to define those 99th percentile limits.^8^ These examples highlight that the disease states are a continuum with health – with a proverbial line in the sand often being drawn at the point where the existing evidence shows a significant risk.

Clinical decision limits should ideally be derived from evidence-based studies however; many that are currently in routine use in laboratories may be historical and based on expert or consensus opinion.^5^ Clinical studies may determine specific CDLs for a test to predict or diagnose disease.^9^ That being said laboratorians review both RIs and CDLs on an ongoing basis. This is a process of quality improvement to update these values based on the latest evidence-based literature and/or recommended guidelines.

Both RIs and CDLs are also dependent on the analytical method used to derive them and may not be interchangeable across different methodologies or analytical platforms. Hence, these need to be verified by the laboratory for their analytical method. Since the early 2000s, there have been substantial moves to harmonise routine biochemistry assays with one of the expectations being that this would also allow for common RIs and CDLs. While, progress has been made in this area, the lack of assay harmonisation remains a considerable obstacle.^5^

## Biological variation and analytical variation

### Biological variation

Another important concept to understand the factors that influence the number generated by a test is the concept of biological variation (BV). Biological variation refers to variability in the concentration or activity of the substance being measured (measurand) around a homeostatic set point. This variability is because of a host of innate physiological factors within an individual and may display predictable daily, monthly, or seasonal biological rhythms. Different individuals may have different set points.^10,11^

### Analytical variation

The analytical variation (AV) represents the imprecision of the test assay because of differences in testing methods and equipment. It is important to acknowledge that there can be variations even in the same sample run by the same instrument at close time intervals, as all analytical techniques have inherent random variation.^12^ This imprecision is expressed as the coefficient of variation of the analyser (CVa). Coefficient of variation of the analyser is a metric that labs should be monitoring, as there are guidelines for most assays with regard to what is desirable, optimal, and minimal allowable CVa.

The uncertainty around the true value of a laboratory result is a combination of the BV within that individual and the CVa of the test method within that specific laboratory. This combination of BV and AV is important to quantify so that one is able to assess whether a change in a patient’s results over time represents true pathology or just ‘background noise’. All ISO 15189-accredited medical laboratories are required to determine the uncertainty associated with all their quantitative assays and have these available to clinicians and laboratory users at their request.^13^ It is likely that the vast majority of clinicians are not aware of this measure to assess the uncertainty of a test result, or that they can request this information from their local laboratory.

Another way of assessing if a change in a patient’s result represents a true change in condition or response to management is to determine the reference change value. Laboratories should be able to provide this information to the clinical team and online calculators are available (however, the lab would still need to provide the CVa information).^14^

Ideally, every individual would have a ‘biological passport’ with personalised RIs (pRI) that have been calculated using the individual’s previous test results obtained in a steady-state situation. These would be continually updated as new results become available and be reported with each result. Currently, this is not practical or possible in most laboratories because of a lack of adequate lab and hospital information systems and electronic health records. The pRI is dependent on the accumulation of an adequate number of results (depending on the model utilised) in the relatively well individual.^15^ This means that the individual would have adequate access to healthcare facilities, laboratory services especially for wellness testing/screening, which is not always possible, particularly in resource-limited settings.

The boundaries between health and pathology are grey zones, while clinical medicine often demands clearer delineation. There is no one true value – a single laboratory result represents a range of possible values. The laboratories’ imperative is to keep that range as limited as possible to ensure the clinical utility of the test. Clinicians are also required to demonstrate some level of flexibility in the interpretation of test results in addition to reviewing against the combination of the prevalence of the disease in question and the history and clinical features of the individual. Much work is still required to overcome the complexities surrounding establishment of RIs and CDLs.
